# Right by your side? – the relational scope of health and wellbeing as congruence, complement and coincidence

**DOI:** 10.1080/17482631.2021.1927482

**Published:** 2021-06-07

**Authors:** Pelle Pelters

**Affiliations:** School of Health & Welfare, Halmstad University, Halmstad, Sweden; Department of Education, University of Stockholm, Stockholm, Sweden

**Keywords:** Health, well-being, concept relation, hermeneutics, normativity, Sweden

## Abstract

**Purpose**: Although the relation between health and well-being is deemed conceptually important, it is diverse and intractable. The aim of this small-scale study is to reveal different possible relations of the concepts of health and well-being, interrelation of these relations and consequences of implied normative expectations in the relations.

**Method**: Primary data originate from course literature in Swedish health education. Additional data included scientific articles and website content (collected from WHO and via Google) and were analysed with objective hermeneutics.

**Results**: Congruent, complementary and coincident relations were found. In congruence, health and well-being are synonyms. Complement relations contain: “quality” with well-being as overall aim, “plurality” with health as umbrella term, “well-being as positive health”, “enhancement” with health and well-being potentially boosting each other and “subjectivity/objectivity” with objective health complemented by subjective well-being. In coincidence, health and well-being are counter-intuitively regarded unlinked, which may challenge expectations concerning health promotive activities. Independent and affiliated relations were identified.

**Conclusion**: In congruence and complement, health and well-being are mostly aligned whereas in coincidence, their quality may be decoupled. In the discursive climate of second modernity, the relation of health and well-being tends to conflict and ambiguous coincidence, demanding ambiguity tolerance as key skill.

## Introduction

There is undoubtedly an expectation of an “intimate connection” (Brülde & Tengland, 2003, p. 238) between health and well-being, a connection that is present in lay persons’ opinions, e.g., when well-being is mostly associated with the term health (Durón-Ramos et al., 2019; see even Pons-Vigués et al., 2017). That link between well-being and health is even scientifically deemed both conceptually important and an intimate one within a health discourse that is hardly imaginable without this interlinkage (Brülde, 2000; Kingma et al., 2011). But despite its importance and widespread use, the relation between health and well-being is far from clear-cut. It rather is diverse and complex and has even been called intractable (Kingma et al., 2011). This intractable fuzziness of the relation between health and well-being can be recognized in a variety of phenomena, of which the following are mere examples: For one thing, it is habitual (though not universal) practice to mention the two concepts of health and well-being in the same breath without them being taken into consideration or having any bearing on the topic at hand further into the argument; as can be exemplified by scientific articles whose titles mention “health and well-being” as related (such as Clift et al., 2016; Mactaggart et al., 2016; Shepherd et al., 2016) but do not pursue both concepts as related in the main text. Another example is the emergence of different descriptions of the relation between health and well-being within the same text or in different texts accounted for by the same stakeholder. According to Erk (2011) texts edited by the World Health Organization (WHO) show this phenomenon. Even scholars in the field of medical philosophy dispute over the concept relation (Brülde, 2000; Nordenfelt, 1987; Tengland, 2007, please note that “concept relation” is used as a short form of “the relation of health and wellbeing”). It is moreover noteworthy that the relation of these concepts as a topic of its own are very rarely explicitly addressed in textbooks (see Hallberg, 2010; Hanson, 2004; Pellmer et al., 2012; Scriven, 2013 for missing discussions; Brülde & Tengland, 2003 being an exception), despite its discursive and practical significance. Finally, as Cameron et al. (2006) mention, both health and well-being are broad, complex and ambiguous concepts that often work as taken-for-granted catch-all terms. As such, they may create confusion regarding their meaning individually and, all the more, their combined conceptual meaning and relation that is rarely defined in detail (cf. even McLeod & Wright, 2016).

It is this significance of the health-well-being relation combined with its implicit treatment and a fuzzy, shifting understanding of the relationship that turns the concept relation between health and well-being into something which is hard to grasp, yet important to know. This is especially true when conducting health and well-being focusing activities as unawareness of how the concept relation is understood may affect results in unforeseen and possibly unfavourable ways (see even Dooris et al., 2018). There are two process-related arguments for raising awareness for the health-wellbeing-relation and their interrelation with intended activity outcomes. First, there is a recent trend in public health to increasingly use well-being as an explicit core concept (not merely a backbone) in health promotive interventions (Dooris et al., 2018) and the number of stakeholders feeling obliged to join into the preparation phase of that health promotive work, all on the basis of their very own understanding of health and well-being, is on the rise as well (Cameron et al., 2006; McLeod & Wright, 2016). That concerns planning the actual activity/intervention, organizing resources for it and creating (political) spaces of possibility for their realization. Hence, raising awareness for the potential range of concept relations appears imperative as a condition for focusing on the aimed-for phenomenon and relation as well as achieving the means for its realization, whatever the intended outcome. Second, not clarifying the implied relation may present a semantic as well as an ideological barrier during the intervention as well. These barriers are assumed to have the potential to result in misunderstandings and breaches in communication (Agarwal, 2010) which may contribute to, for example, not reaching the target group of an intervention (Björklund, 2008) and/or reproducing this groups’ understanding or courses of action (Broom, 2008). Educators (or other health professionals) are, third, in this scenario expected to be professional representatives of science, knowledge and even health. That may not always be entirely true as studies on health students’ general health literacy and health professionals’ competences hint at (e.g., Elsborg et al., 2017; Naccarella et al., 2016) and calls for an inquiry.

Conducting unsuccessful interventions in the wake of an undefined relation between health and well-being might, moreover, contribute to an understanding of certain groups as “unteachable” by educators (Rowley et al., 2014), which, consequentially, may confirm and even widen the gap between “them” as the deviant “Other” and “us” as representatives of the normal, responsible majority. Hence, chances are that interventions may contribute to a debilitating labelling process (Becker, 1966). Similarly, the theoretical approaches chosen while developing and administering interventions, policies, etc., that deal with health and well-being represent choices about that which is desirable with probable disciplinary and regulatory consequences. Here, a notion of normativity, of the expected (and otherwise sanctioned) and the common as the “normal” case (Johnson, 2000), comes to the fore that can be referred to the workings of biopower (Foucault, 1990). These notions show not only in how health and well-being are assumed to be understood (cf. Dooris et al., 2018) but also concerning their relation to one another. Often, an alignment between the quality of health and well-being, i.e., good health corresponds to high well-being, seems to be intuitively correct and is presumed (cf. Brülde & Tengland, 2003). That is, however, a doubtful hypothesis and needs to be investigated—as needs normative implications of the health-wellbeing relation in general.

The diverse, yet fuzzy understandings of the relation between health and well-being and its possible consequences call for a clarifying overview as a reflexive tool for all kinds of health practitioners and other health professionals, which motivates why the result of the present study is needed. Following in the footsteps of Paulo Freire (1973) and his focus on “conscientisation”, i.e., raising critical awareness and problematizing in dialogue as a first step of change, such a reflective tool is deemed to be used in a variety of ways to mitigate the fuzziness of the concept relation: This tool would provide a means for clarifying which version of the relation is assumed in the development and conduction of practical interventions, in organizational standpoints as well as in conceptual, legislative or other negotiations in health (care) policy. This may help decision-making while planning and organizing interventions. The clarification process may also include raising awareness for different interpretations of the relation and their implications as well as problematizing the co-existence of different versions of the relation in any context that strives at transmitting clear messages like ongoing interventions with different target groups. As this may improve all education occasions, which address the topics of health and well-being (as related to each other), even university-level education for health professionals-to-be could be targeted. Then, students could be provided with a comprehensive overview in a way that is not given today (according to my own experience and personal communications with colleagues). That should allow them to gain clarity of their own point of view and that of those they meet as well as reflect upon the benefits and costs of different relations between health and well-being in order to being able to apply them from a knowledgeable, less biased, reflected position.

Moreover, the overview may facilitate targeting the issue of the normative load of concept relations in a norm-critical fashion (Björkman & Bromseth, 2019; Kumashiro, 2002; Pelters, 2018). Building on the Freirean approach, norm-critics highlight latent norms as self-evident, tacit expectations that often make themselves felt as “intuitively right” or “counter-intuitive” ways of behaving, understanding, feeling and appearing. This approach may improve achieving intended ethical guidelines and values by applying a critical stance towards one’s own views and expectations and by becoming aware achieving the chance to broaden the norm spectrum. A norm-critical stance is connected to different categorial orders of power and highlights processes of othering, is based on a Foucauldian view on power (Foucault, 1990), and investigates powerful narratives within societal discourses, such as the health discourse.

The present-day health discourse (in all its variety) in second modernity (Giddens, 1991) represents the background of this study. It occupies a powerful position in Western societies, in which it permeates all sectors of the hence so-called “health society” (Kickbusch, 2007, p. 144). Here, health is constructed as a “monolithic, universal good” (Metzl, 2010, p. 9) and a “super value” (Crawford, 1980, p. 379). The underlying hegemonic view on health, the biomedical paradigm, is characterized by a biomedical, technoscientific interpretation of one’s health status grounded in a logic of self-evaluation, self-care and self-regulation towards achievement of an—in principle unlimited—personal health potential promotion (see, e.g., Nettleton, 2013, conceptually discussed of Seedhouse., 2001). That health discourse clearly maintains and supports the neoliberal logic of individual choice, responsibility and risk management engrained in many health-promotion and preventive efforts (Ayo, 2012), despite the emergence of other approaches to health/work (such as salutogenesis, Antonovsky, 1987) and the rising importance of well-being in public health (Dooris et al., 2018). The latter indicates a discursive convergence of health and well-being, as does the health norm of well-being (stating that people are supposed to feel good in the cause of their health-related activities and due to their health commitment, Pelters, 2012) or descriptions of discursive characteristics of well-being (e.g., expressing its positive connotation by addressing well-being as positive health, Ganesh & McAllum, 2010).

The health discourse abounds with normative demands and expectations, pointing at the moral imperative of health (Lupton, 1995) and the notion of healthy bio-citizens as virtuous, solidary members of the health society (Halse, 2009). Being a good bio-citizen requires a “preoccupation with personal health as a primary (…) focus for the definition and achievement of well-being” (Crawford, 1980, p. 368] called this attitude “healthism”). In connection with this attitude, people’s moralizing judgements of their own and other people’s health and health practice (see, e.g., Crawford, 2006) appear justified. These morally driven judgements may contribute to processes of blaming the victim, othering and stigmatization (Guttman & Ressler, 2001; Mensinger & Meadows, 2017). The achievement of health is, however, complicated by the array of competing and contradicting sources, needs and demands from which subjects are mandated to choose (Kristensen et al., 2016). Among these, the paradoxical requirements for both control and relieve, productivity and consumption (Crawford, 2000) represent only one challenge.

In this discursive climate, I deem both the array of intelligible relations of health and well-being as well as their potential normative load and implications as in need of clarification in order to explicitly create a reflexive means for future usage clarification, impact assessment, guideline realization, further concept development and last but not least education. This is considered to mitigate the intractability of the relation through the ability to name, observe, reflect on, compare, use and develop it, on all societal levels from intention to achievement. Such an overview over the status quo of understanding the relation of health and well-being and its normative implications does not appear to exist to date, to my knowledge.

## Aim

The aim of this small-scale study is to understand and synoptically compile the different relations of the concepts of health and well-being, by analysing its implicit and explicit presentation in different forms of texts that are routinely made available to Swedish health education students.

Research questions comprise:

1. What different understandings of relations of health and well-being are presented?
2. How are the different understandings of relations of health and well-being interrelated?
3. Which of those relations are presented as seemingly “counter-intuitive” or “regular”?
4. What challenges could in particular seemingly counter-intuitive or deviant relations imply for people’s understanding of health and well-being?

While the first two questions, focusing on understandings, will be answered in the results section, the latter two, focusing on normativity, are taken on in the discussion.

## Method

### Design

As the study aims at understanding a concept relation as implicitly and explicitly expressed by the use of the related concepts in different forms of texts, a qualitative, hermeneutical study design is chosen (Lamnek & Krell, 2016).

In particular, the sociology-based structuralist approach of Oevermann (2002) is used, which aims at uncovering different ways of dealing with a challenge or solving a task. In this study, the task is relating health and well-being. The different ways of handling such tasks that emerge from the data are regarded as cases of social practice, which are deemed socially acceptable, as they otherwise would not be detectable. Here, the different detectable cases are the different ways of relating the two concepts, which are the different social accepted possibilities people have to understand the relation between health and wellbeing in this specific moment in time in western societies. Compiling these options is what the study intends to do. Hence, this approach is deemed suitable.

Moreover, the specific case in the data does always simultaneously represent a general case within the chosen social context. Hence, cases are at the same time specific and general. Both the social acceptability and the simultaneousness of general and specific cases are basic assumptions (Oevermann, 2002). Applying this structuralist notion in data collection promotes the purpose of finding maximum case variations of the health-wellbeing-relation, even in small-scale studies (Silverman, 2013). As the number of socially acceptable cases is limited (Oevermann, 2002), the ambition to find all of them is not understood as a contradiction to performing a small-scale study.

It is noteworthy that the social acceptability, however, does neither translate into an appreciation of a practice nor gives any account of its dissemination, but simply conveys that these varied ways of dealing with the task (these different cases) are possible and correct in a certain social context, time and place. As this approach targets the whole spectrum of different socially correct, unquestioned and accepted ways of acting and understanding in a certain social environment, it applies the notion of structural, not empirical-statistical generalization (Oevermann, 2002).

### Data collection

#### The selection of data source

The selection of data sources is motivated by the study aim to synoptically compile the different options of understanding the relations of the concepts of health and well-being for members of Western societies. As a representative of western societies Sweden is chosen. Sweden is deemed suitable as the country is highly focused on individual self-expression and secular-rational values (cf. World Value Survey, 2017). Therefore, the cultural background appears to be in line with attitudes that are expected to guide people’s health-awareness and productivity, unsuspicious of being affected by prejudices or misconceptions (Crawford, 2006). As its representative members, health education students are chosen. One reason for choosing the situation of a Swedish health education student is the fact that I am teaching those students and have access to relevant data. Another more important reason is that these students are regarded as good representatives of health-aware people due to the expectation that they are characterized by a considerable interest for health as a topic and practice as well as a curiosity to resolve the lack of information to become a professional. Here, the question is what information the group is provided with—which basically is the very question for anyone looking for orientation in this field.

As students are supposed to become knowledgeable in their field of expertise, the spectrum of sources health education students may have access to should provide a good overview of the possibilities of understanding this relation and thus suitable data to synoptically compile those options. The collected data focused hence on sources that are routinely made available to Swedish health education students in such a way that with the commencement of their studies, students are introduced to (mostly Swedish) course literature. Moreover, they are often referred to (English) publications of the WHO and to scientific databases for further information. In addition, students may gain easier, extended access to other resources on the internet, due to their student account, which they could make use of by Google searches in Swedish. All of these forms of texts are potential, yet customary sources of information for Swedish health education students.

As students have a guaranteed access to course literature as literature that is supposed to provide a representative overview over important concepts in one’s field of study, these sources are regarded primary data whereas secondary data may rather be potentially accessed, i.e., may or may not be taken into consideration. However, the context of studies in health education may be limited. Therefore, secondary data sources are also included as a precaution to minimize the risk of missing other versions of the relation between health and well-being and a consolidation of the findings in primary data sources. The secondary collection process has been inspired by scoping reviews (Arksey & O’Malley, 2005) as “a process of mapping the existing literature or evidence base” (Armstrong et al., 2011, p. 147) for available key concepts and research findings in a rapid process and may take into consideration both scientific and non-scientific data to broaden the data base. Hence, this choice of data sources maximizes chances to highlight as many versions of the health-wellbeing relation as possible and meet the goal of providing a synoptic compilation of accessible understandings of the relations between health and well-being.

#### The data collection process

Data collection has been conducted in two steps. A first data collection took place in October 2016 to January 2017 including course literature (primary data) as well as databases, WHO and googling the internet (secondary data). A second data collection was conducted in March 2020 in order to boost the volume of considered textbooks and articles related to university-level health education (primary data). Also, a second database search (secondary data) was conducted, to update and add rigour to the study by means of applying more systematic search terms.

The data collection and selection process is described in Table I, see supplementary material for more details about the selection.

**Table I.** The process of data collection and selection

Collection round no.	Primary/secondary data source	Data	Captured by data	Initial selection	Second selection	Final selection
1	Primary	Course literature	Sound, established scientific knowledge	18		5
	Secondary	Scientific databases	Scientific studies and negotiations	793 (≤ screened by title)	43 (≤ screened by abstract)	5
		WHO	Important international stakeholder	3930 (≤ first 200 chosen)	200 (≤ screened by title and text excerpts)	10
		Google	Lay persons as stakeholders	454 000 (wb + food) 239 000 (wb + exercise) (≤ first 100 chosen)	200 (≤ screened by title & text excerpts)	8
2	Primary	Course literature	Sound, established scientific knowledge	29		4
	Secondary	Scientific Databases	Scientific studies and negotiations	625 (≤ screened by title)	51 (≤ screened by abstract)	17

#### The different data sources and their contribution

##### Course literature (primary data)

The course literature include books and articles which are used as introductory, first-term course literature in two Swedish bachelor programmes in health education, a term during which basic concepts are in focus. Here, those health-wellbeing relations show that are marked important, sound and established enough to be presented to first-term students. Publications included in the search represented a) comprehensive introductory texts to b) theories of health, public health, health promotion, health identities and health education which c) introduced the concepts of health and well-being. Exclusion criteria were literature on a) specific topics (for example, empowerment or obesity), b) epidemiology (when not explicitly dealing with issues of health and well-being) or c) methods and methodology. In the two rounds, a total of seven textbooks (one in English, six in Swedish) and two Swedish articles represented the final sample, which included conceptual investigations and introductions regarding health and disease, public health, health promotion, health and lifestyle as well as health identities. At this stage, the described concept relations were named and outlined in the first round while a new relation could be outlined in data collection round no. 2.

##### Secondary data sources

- *Databases (scientific literature)*: During the two data collections, three databases each were searched to understand the state of research and scientific discussion regarding the relation between health and well-being. During the first collection, PubMed, Web of Science and OneSearch were searched using the search terms “relation between health and well-being”; “relation between well-being and health”; “connection between health and well-being”; “connection between well-being and health” as well as “health AND well-being AND ‘concept relation’”. In collection round no. 2, besides PubMed and Web of Science, Academic Search Premier replaced OneSearch, due to accessibility issues. The following keywords were used (summarized here as a Boolean search mode): i) Health AND ii) “well-being” Or “well being” OR “wellbeing” AND iii) “concept analysis” OR “concept relation” OR “concept definition” OR “theoretical concept”. The final sample contained one practical viewpoint discussing the health concept, one review of the concept of well-being discussing its link to health and different areas of life, a commentary on the challenges to the spirit of WHO’s Ottawa Charter discussing disabled persons rights, one conference report presenting a workshop on concepts of health and disease and one doctoral thesis discussing health as a human right, four studies using empirical input for a theoretical aim, three critical discussions of practices and ten concept analysis and discussions. During both data collections, previously established relations were confirmed and illustrated, although no new relations were reconstructed.
- *WHO (indirect stakeholder consultation*, Arksey & O’Malley, 2005): The internet presentation of the WHO was searched for texts linking health and well-being in order to understand if the most important stakeholder on the health market and provider of one of the most cited health definitions conceptualizes the health-wellbeing relation in an additional way. The global sites on www.who.int have been searched using the search terms “‘health’ AND ‘well-being’”. Ten hits were selected as probably describing a new relation. Among those sites were publications, events and fact-sheets. However, no new relations were found after reviewing the hits as a whole.
- *Google search engine (indirect online “common people” stakeholder consultation*, Arksey & O’Malley, 2005): A Google-search for the relation between well-being and two major health promoting practices, i.e., determinants of health (food and exercise) has been performed. The search was performed in Swedish using the search terms “well-being + food” (välbefinnande + mat) and “well-being + exercise” (välbefinnande + träning). Targeting food and exercise is conditioned by their high status as *the* two most relevant health-promoting practices emerging from the so-called obesity crises (Quennerstedt et al., 2010). As such, food and exercise appear as valid representatives of health against the background of the construction of health as a performatively staged reality and identity in terms of *doing health* (Pelters, 2012). This notion is confirmed by studies on lay persons’ understandings of health (Blaxter, 2010; Flick, 2000) in which health has been described as a lifestyle and the practice of living healthfully. Eight hits could be identified as possibly containing new forms of the relation. Among these were website of county councils, health bloggers, companies offering health promotive activities and health magazines. One new case of relation could finally be identified on a website that belonged to a health magazine.

### Analysis

A combination of inductive objective hermeneutics and deductive contrasting was used to find new relations between health and well-being. Objective hermeneutics is a sequential analytic method developed by Oevermann (2002) guided by a central question: According to what underlying assumption (here: regarding the relation of health and well-being) does it make sense to build up an argument (assemble the text) in the way the text does? It involves sequentially hypothesizing about the latent assumption and testing these hypotheses while revealing the text bit by bit, thus following how the argument is build up in the text. The method is very close to the text in that it analyses what is written, not what is assumed that the author wants to tell or how the text may relate to pre-defined concepts. It works even with small text units such as parts of a sentence and is thus suited for the small text fractions delivered by search engines as well. Deductive contrasting refers to using the list provided by the first analytic step as a deductive template for record exclusion during data collection.

Deductive contrasting and objective hermeneutics have been integrated as follows in the study during the first data collection round: After the course literature screening was finished, a first *inductive analytic step* was conducted using objective hermeneutics, i.e., a hypothesis was formulated about what implicitly assumed relation between health and well-being is revealed by how the text is composed. That hypothesis was compared to variations in the relation emanating from the analysis of earlier records (if existing). If the text was regarded matching one of those variations, the data record was discarded. If the text appeared to represent a new way of interlinking health and well-being, the data record was read on and—if confirmed as new—that assumption was added to the list of existing relational variations. This procedure resulted in a first list of possible relations between health and well-being. The list was then used as a deductive template in further analytic steps (by comparison to new data) and assembled further during the course of analysing database, WHO and Google hits. The analytic process was thus intertwined with the search and screening process. The comparative procedure of deductive contrasting was repeated during the data update with regard to course literature and scientific articles in 2020.

### Ethical statement

The author declares that this research has been conducted in an ethical and responsible manner following the recommendations of the Swedish research council (All European Academies [ALLEA], 2018). As only publicly accessible data is used as empirical data (in terms of textbooks, scientific articles and website content), approval of the local ethics review committee was neither deemed necessary nor obtained, however.

### Theoretical approach for discussion

A detailed theory by theory investigation of health would exceed the scope of this paper. However, scholars from different Western countries (Blaxter, 2010; Brülde & Tengland, 2003; Franke, 2012; cf. Seedhouse., 2001) agree on defining health as functionality, as balance or as the absence of disease (albeit they all add different other understandings). These three understandings of health are considered common enough to represent relevant concepts for discussing the results of this study. Moreover, these understandings parallel the classical description of lay people’s understandings of health as (disease) vacuum, reserve and equilibrium (Herzlich, 1973) which were confirmed (but also in part expanded) by more recent studies (Blaxter, 2010; Flick, 2000). Drawing on the abovementioned authors (Blaxter, 2010; Brülde & Tengland, 2003; Franke, 2012; Herzlich, 1973; Seedhouse., 2001), the three notions of health are understood as following:

- Health as *the absence of disease* describes the notion that they who are not diseased and not biologically disadvantaged are healthy. This definition is connected to certain general unawareness of the body, which only attracts attention when signs of ailment or disease occur. It includes a dichotomy between health and disease, i.e., the two concepts are mutually exclusive, i.e., a person can only be either or at the same time.
- Health as *functionality* describes a physical strength or robustness towards bodily challenges (close to fitness) as well as mental and other capacities that enable people to perform their tasks in private and public. Those who are capable and competent enough to fulfil norms and role expectations as well as cope with everyday life challenges and achieve what Nordenfelt (1987) called vital goals in life are considered healthy whereas a movement towards the opposite is a movement towards disease.
- Health as *balance*, finally, depicts a health characterized by harmony and feeling well connected to a high quality of life. Health may here be characterized by a balance between outer and inner factors or focused on inner factors alone. A person in a stable, quiet, well-adjusted and well-tempered state is considered healthy. Even here, a movement towards imbalance often represents a development towards disease.

Another common definition of health is, hardly surprising, well-being, although not every author mentions this definition (Blaxter, 2010 is the exception here). However, as these definitions will be used to discuss the implications of different relations of health and well-being concerning understandings of health, doing so would seem tautologous and circular regarding health as well-being as it rather is a part of the data. This definition will thus not be considered.

## Results

### Relations

Three different major relations between health and well-being have been identified—congruence, complement and coincidence—of which the latter two relations include different versions (see overview in Table II,III). A third table with a detailed overview of the different data sources and the relations, which are depicted in each of them, can be found in the supplemental material.

**Table II.** Overview over different relations between health and well-being

Overall relation	Versions	Description
Congruence	-	Health = well-being
Complement	Quality	Well-being overall aim
	Plurality	Health as umbrella term
	Enhancement	Impact: health & well-being boosting each other
	Subjectivity/objectivity	Health = objective + well-being = subjective
	Well-being = positive health	Well-being as representative of positive health
Coincidence	Autonomously confirming/conflicting effects	Independent: joined or antagonist developments between well-being & health as unlinked concepts

#### Congruence: well-being = health

In their traditional definition, the WHO calls health “a state of complete physical mental and social wellbeing” (1948, in Erk, 2011). Thus, one abstract concept—health—is explained by using another abstract concept—well-being—that sets an equal sign between the two of them, as Erk (2011) points out. Some other sources (Hanson, 2004; Korp, 2016; Pellmer et al., 2012; Scriven, 2013; www.who.int) use the definition as well and thus allow for an interpretation as synonyms. It should however be noted that this way of interpreting the WHO definition does not stand unquestioned. An impression of a certain conceptual inconsequence and interpretational fuzziness on the part of the WHO occurs as the latent content of the sentence may be interpreted differently in contrast to its manifest content which allows for health to be understood as the umbrella term (see plurality version).

Different expressions of a synonymic understanding could also be found in other publications. Hallberg (2010) mentions congruence as a usual understanding in everyday life, something which is confirmed in other articles (e.g., Dooris et al., 2018; Pons-Vigués et al., 2017). Moreover, two-thirds of the articles collected in 2020 expressed an interchangeable use by, e.g., referring to well-being and health as each other’s “proxy” (Aldrich, 2011, p. 95), “surrogate” (Agenor et al., 2017, p. 919), “shorthand” (McLeod & Wright, 2016, p. 777) or “tag” (Cameron et al., 2006, p. 349) or other ways to express congruence (Boers & Cruz Jentoft, 2015; Martin & Woodgate, 2020; Modise & Johannes, 2016). Brülde and Tengland (2003) refer to Gadamer’s view on health and well-being in a way that has a strong resemblance with conceptual congruence. Finally, Grundmann (2014, p. 555) finds a use of well-being that is “akin to the modern idea of health” in certain biblical writings as well. This kind of relation is hence a very prominent one.

#### Complement: well-being + health

In this section, a variety of understandings of the health-wellbeing relation are revised that have the assumption in common that health and well-being are (at least in part) different concepts that share a direct relation.

##### Quality

This relation is characterized by regarding wellbeing as an umbrella that includes health and thus constitutes a hierarchy between the two. Going back to the WHO-definition of health (1948, in Erk, 2011) for example, an alternative reading that elevates the status of well-being starts off by understanding health as a resource, a term implying that health is a prerequisite and means to a different end. An example of that end is represented by the Ottawa-Charter (World Health Organization [WHO], 1986) stating, e.g., “health promotion is not just the responsibility of the health sector, but goes beyond healthy life-styles to well-being”, with well-being appearing as a final destination of the health journey (WHO, 2012). Here, health takes on the role of a determinant of well-being, i.e., a piece of the puzzle that is well-being, with well-being being something bigger and more comprehensive, as is also apparent in the Canadian Index of well-being (University of Waterloo, unknown) and other web resources found in the data.

Considering well-being a more overarching principle compared to health is a point of view that has been confirmed in most comprehensive (text-)books (Brülde & Tengland, 2003; Erk, 2011; Hallberg, 2010; Hanson, 2004), even if its relevance as a useful approach in the health field has subsequently been critically discussed, even trimmed “to focus on health-related well-being and not well-being in general” (Vilhelmsson, 2014, p. 66, cf. Korp, 2016). Moreover, a great deal of the scientific articles that focus on well-being relates health and well-being according to the quality relation (Aldrich, 2011; Dooris et al. 2017; Konu & Rimpelä, 2002; Kahn & Juster, 2002; Kimiecik, 2011; Kingma et al., 2011; Martin & Woodgate, 2020; Sointu, 2006; Wolbring, 2006). The minor role of health in this relation shows, e.g., in a quote from Sointu (2006, p. 335): “physiological health is conceptualised as a part of a more wide-reaching notion of wellbeing. [It] is seen to touch all areas of life, ultimately manifesting (…) as a sense of ‘natural’ contentment, fulfilment and harmony”. In addition, well-being appears in the quality relation often to be understood according to an eudaimonic notion, i.e., a focus on meaning, self-realization and complete functionality (implied in Ryan & Deci, 2001; Tončić & Anić, 2015, spelled out in Dooris et al. 2017; Kimiecik, 2011). Its pursuit is directed into the future, towards a better quality, while the actual health and wellbeing here and now usually appear as flawed.

##### Plurality

Here, health may be understood as the umbrella term, an overall, broader issue, which is either determined or defined by a range of different factors with well-being being one of them. This relation represents hence the opposite of the aforementioned. What is subsumed under the health umbrella may, however, vary, as does the role well-being plays with regard to health as either causal or conceptually contributing. These two may be regarded as different perspectives within the plurality relation.

The 1948 WHO-definition of health, which may alternatively be interpreted as representing plurality, includes a division of well-being into physical, social and mental components which internally complement each other as determinants of health (Wolbring, 2006). The same dimensions appear even in Boers and Cruz Jentoft (2015) who have a view on health as a capacity to cope, which resonates with Nordenfelt’s definition of health (Nordenfelt, 1987) in which well-being plays a causal, health-determining role. The range of included determining dimensions can be expanded beyond the bio-psycho-social model of health to include more dimensions such as “occupational, intellectual, spiritual and environmental” (Pons-Vigués et al., 2017, p. 7). That resonates with holistic models of health and health promotion mentioned in a public health context, which include a whole array of factors influencing health in the shape of determinants (Erk, 2011; Hanson, 2004; Korp, 2016; Pellmer et al., 2012; Scriven, 2013; www.who.int). When it comes to articles, the plurality relation often invoked in papers addressing the overall topic of health as frame of reference (Boers & Cruz Jentoft, 2015; Burkert et al., 2015; Jaberi et al., 2019; Pons-Vigués et al., 2017; Svalastog et al., 2017), comparable to the focus on well-being in articles representing the quality relation.

Moving from determination to definition, the conceptual contribution of well-being is represented in Brülde’s (2000) multifactorial concept of health. Here, health is the umbrella term for a combination of conceptually relevant biological, functional and well-being dimensions—a strategy, which is also pursued by Vilhelmsson (2014). Another version of that conceptual perspective has been described by Tengland’s (2007, p. 257) two-dimensional theory of health, in which well-being in terms of experienced “positive moods and sensations” is joined by developed and usable “abilities and dispositions that members of one’s culture typically develop” as health defining aspect. No matter, however, if plurality refers to causal or conceptual contributions, the relation between health and well-being is in any case a hierarchical one, with health being “bigger” than and inclusive of well-being.

#### Enhancement (unidirectional/bidirectional)

In this relation between health and well-being, the time frame indicates succession, as the enhancement relation describes impacts that health and well-being may have on each other. This is a causal relation with health and well-being representing non-overlapping determinants and concepts. The impact may either work unidirectional, i.e., one of them (health or wellbeing) impacts on the other, or bidirectional, i.e., health and well-being influence each other reciprocally. The unidirectional enhancement of health due to a promoting influence of well-being can by exemplified by Quennerstedt (2007) who emphasized the contribution of “learning, democracy and well-being in ongoing movement activities as potentially health-developing” (see even Agenor et al., 2017; Howell et al., 2007). The other way around, the enhancing impact of health on well-being becomes clear when bidirectional enhancement is presented. Here, health-related self-conquest and activities contribute to (hedonistic and/or eudaimonic) well-being in terms of self-realization or positive feelings which then again may boost health(y) behaviours or health outcomes as a commitment towards being fully functioning. That direct interrelation may be based on suppressing one’s spontaneous needs and is linked to subjective experiences. The following quote provides an example:

“Cycling is truly dull exercising to me and even though I think you mostly should practice fun stuff, I think that sometimes exercises need to be boring as well. (…) When I was done with that morning’s spinning half an hour later, I was sweaty, happy and proud. I had had time to exercise even though I fell asleep again and I had overcome my negative attitude towards biking in particular. Best feeling!” (Axelsson, 2015)

In the quote, spinning is presented as an unloved, yet necessary health activity (representing health, see rationale for using a Google search as data source) that, once completed, turns into a source of well-being and joy, an experience which then again sows the seeds for further spinning. That mutual dynamic process of bidirectional, reciprocal enhancement has been very prominent on the internet (Google-search results).

In a more “basic” version, bidirectional enhancement can even be observed in some of the scientific articles (Jaberi et al., 2019; Kimiecik, 2011; Ryff, 2014), in textbooks (Brülde & Tengland, 2003; Hanson, 2004) and on the WHO website.

##### Subjectivity/objectivity

In a fourth version of the complement relation, the motives of subjectivity and objectivity are essential. Well-being appears as the perception of a self-referred, experience-based, often mind-related quality of “feeling” on the part of the individual subject and health as the quantifiable, diagnosable, medical view of “materiality” represented by the professionals in biomedicine (Ryff, 2014). Those two notions may be combined in variable ways, turning them into potentially equal positions as two sides of a coin, without referring to a potential causality or conceptuality within this concept relation.

The subjectivity/objectivity relation is mentioned in most teaching materials (except Pellmer et al., 2012 and Scriven, 2013), the majority of articles (Agenor et al., 2017; Bergland & Kirkevold, 2001; Brülde, 2000; Burkert et al., 2015; Grundmann, 2014; Jaberi et al., 2019; Kahn & Juster, 2002; Kimiecik, 2011; Kingma et al., 2011; Sointu, 2006) as well as in the results of the Google-search. An example for the subjectivity/objectivity relation is the presentation of so-called health-cross as mentioned in three references (Hallberg, 2010; Hanson, 2004; Korp, 2016). It describes four compartments which are created by two axes: the spectrum between medical health and disease on a first axis which is crossed in the centre by a second axis that describes feelings of well-being on a continuum between good and bad. Due to the four compartments, the resulting statuses are healthy & well, diseased & unwell, healthy & unwell and diseased & well. The health-cross raises hence awareness for the possibility of combining seemingly contrary, unexpected combinations of subjective feeling and objective materiality but presupposes that health and well-being are inevitably connected. Moreover, those unexpected combinations are presented within a common order of things in which good and bad are both clearly divided and hierarchically classified. Hanson (2004) for instance, points out that certain combinations are to be regarded as second best (sick & well) or in need of avoidance (healthy & unwell), while Hallberg (2010) expresses the unexpectedness of those combinations by introducing them as something that “happens” or “occurs”, as if this was hard to imagine. In presenting versions of the subjectivity/objectivity relation that appear to be aligned or misaligned with expectations regarding the covarying quality of health and well-being, notions of “normal” confirmation and challenging, “counter-intuitive” conflict are created. These versions appear to bear some similarity with those elicited by the coincidence relation.

##### Wellbeing as positive health

Another complement relation is determined by the duality between positivity and negativity and conceptualizes well-being as a representation of the positive side of health. As a number of different versions of it exist, this concept relation rather appears like a receptacle for different connections between well-being and positive (aspects of) health. While traces of it can be found in several scientific articles (e.g., McLeod and Wright (2016, p. 778) description of interventions that “enhance wellbeing in particular areas, such as the use of music in promoting positive mental health” or Burkert et al. (2015) who mention subjective well-being as one positive indicator of health) understanding well-being as positive health is most prominent in the data in Korp (2016). He reviews two different ways of understanding well-being as positive health: on the one hand (with regard to Catford, 1983), well-being may be regarded the optimal value of positive health, its “utmost tip”. That presupposes the use of a gradual continuum ranging from health at its worst (death) through different stages of increased health (e.g., handicap, disease, risk) to health at its best (a.k.a. well-being). This version may be applicable concerning the concept of thriving (Bergland & Kirkevold, 2001) and even more so the concept of flourishing: “Flourishing is described as the optimal state of mental health (…) [Flourishing] is synonymous with a high level of mental well-being, and it epitomizes mental health” (Agenor et al., 2017, p. 915). On the other hand, well-being as the positive side of health may represent the whole range of positive health. This presumes that well-being represents one side of a duality, with the other being “unhealth”, as Korp (2016, p. 60) calls it (without further explanation of what “unhealth” comprises). His text illustrates this take on well-being as positive health by using the above mentioned “health-cross”, explaining that the health-cross is “a model that separates the negative dimension from the positive dimension” (Korp, 2016, p. 60, with reference to Downie et al., 1996). It may thus be claimed that well-being is at least partially understood as positive health, although the concept relation remains fuzzy in the literature and it is hard to define this relation in terms of causality or conceptuality.

#### Coincidence—a non-relation: well-being with or without health

The coincidence relation is technically a non-relation and is best depicted by Korp’s account of Seedhouse’s (2004) description of the conceptual independence between the concepts of health and well-being: “He believes (…) that an individual’s well-being not necessarily has anything at all to do with her ill-health status. Therefore, you have to provide two scales if you want to clarify an individual’s ill-health and well-being status, respectively.” (Korp, 2016, p. 61). To prevent misunderstandings about the mentioned ill-health scale and status: Korp provides an ill-health scale that ranges from not ill to ill and might therefore just as well be called a health-scale. Consequently, the cited statement actually describes the (un-)relation between health and wellbeing. That corresponds to Seedhouse (2004, p. 153) original writing: “the relationship between health and well-being (…) is contingent.”

Especially in the sphere of everyday life (as captured by Google search results) as a realm of doing health, health (understood as practice or lifestyle, Blaxter, 2010; Flick, 2000) and well-being appear to have a flexible, unconnected relation. In other words: Experiencing, practicing or following expectations concerning one concept may or may not result in experiencing, practising or following expectations concerning the other concept. Hence, the dimension of time needs to be considered: Activities that aim at improving well-being here and now may confirm or conflict with practices, which are expected to boost a health potential and promote the realization of health in the future (and vice versa). If there were a connection, causality would be logical to assume, merging this version with enhancement into a more neutral causal concept relation—but it is just that connection that is missing.

As mentioned, there are two potential versions of coincidence, which show up in the Google search results. On the one hand, a *confirming* version may occur when well-being is experienced or realized in, e.g., practicing mindfulness, cooking food from scratch, enjoying a sauna or similar activities that are compatible with what is deemed health-promotive living. Examples of presentations of concept relations that are similar to the confirming version appear in other forums as well (Brülde & Tengland, 2003; Hanson, 2004; Hallberg, 2010; Korp, 2016; Ryff, 2014; www.who.int). On the other hand, well-being may appear as *conflicting* with the demands of health-promotive living. Publishing “13 smart excuses to escape exercise” under the heading “well-being” (Dumanski, 2016) may be regarded as one example. Another one is the existence of so-called comfort food, understood as “foods that people consume in order to attain psychologically comfortable or pleasant states” (Troisi et al., 2015, p. 58). This kind of food usually may consist of potato-chips or other food deemed “unhealthy” from a medical point of view, which is often consumed in large quantities in situations of not feeling well. Well-being is hence achieved by pursuing an unhealthy practice and disconnected from health. This conflicting version could in this explicitness only be observed in the results of the Google-search, although examples resembling conflict could be found elsewhere as well (Bergland & Kirkevold, 2001; Brülde, 2000; Brülde & Tengland, 2003; Hallberg, 2010; Hanson, 2004; Korp, 2016).

### Relations of relations

**Figure 1.**
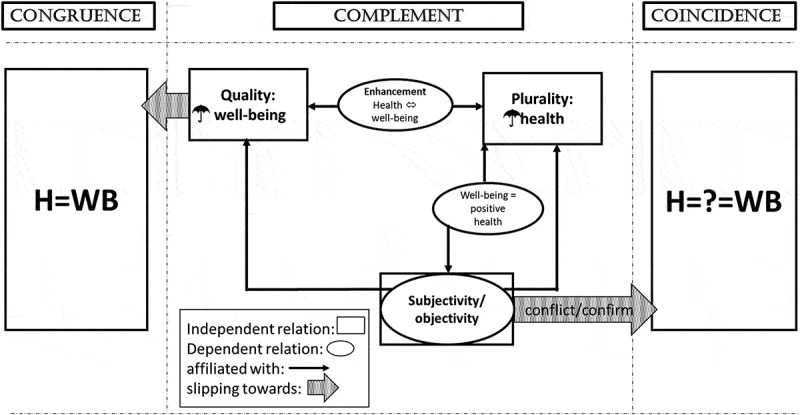
Relations of relations

This section maps the relations between different understandings of the concept relation, in accordance with the data. The result may be called a landscape of understandings concerning the health-wellbeing relation. In-/dependence, overlapping understandings and slippages between different understandings are in focus. The interrelations are illustrated in Figure 1.

In general, different kinds of concepts relations may be differentiated. The concept relations in sub-section A own a model character in such a way that causal or conceptual relations between the concepts of health and well-being may be discussed. Moreover, the different concept relations are allotted the same dignity. The concept relations in sub-section B represent specific versions of the complement relation, in which conceptual overlaps and certain connotation come to the fore. In sub-section C, effects of understanding a concept relation in a certain way are highlighted, which result in slippages between different understandings of the health-wellbeing relation.

#### Independent relations

Congruence represents a common and self-sufficient, independent relation, characterized by synonymity between the concepts. It is most often mentioned in the context of everyday life and lay understandings, although it even emerges in health professional contexts. This relation possesses the same relevance as quality and plurality within the frame of complementing relations.

Judging from the habitual practice to mention the two concepts of health and well-being in the same breath, the complement relation is at the centre of how the relation of health and well-being is understood. Within this major relation, both quality and plurality relations emerge from the data as equally important, with each highlighting on of the concepts as more all-embracing than the other. These two relations appear to be independent, i.e., they cannot be subsumed under other relations (e.g., Martin & Woodgate, 2020; Pons-Vigués et al., 2017). Plurality and quality relation can hence be placed side by side without overlap and also side with congruence.

The possibility of conflicting variations of health and well-being challenges the assumption of an intimate, inevitable link between those concepts. This challenge is articulated and turned into the concept (un-)relation of coincidence. As such, coincidence represents another independent approach concerning the (non-)relation of health and well-being (cf. Seedhouse in Korp, 2016).

#### Connotations and overlaps between different versions of the complement relation

The subjectivity/objectivity relation is more intricate: First of all, it connotes health with objectivity and well-being with subjectivity. Moreover, the subjectivity/objectivity relation may constitute a self-sufficient relation within the realm of complement relations in terms of relating two concepts of equal importance/dignity (as in, e.g., James & Hockey, 2007), while it is also compatible with presentations according to plurality (e.g., Brülde, 2000) as well as quality (e.g., Sointu, 2006). On the one hand, subjectivity/objectivity is hence characterized by a potential connectivity, without, however, being dependent on such a connection as the data do not appear to presume that other concept relation and may as well stand alone. On the other hand, as subjective well-being and objective health are regarded as two sides of a coin, both are necessary to gain something complete, which is bigger than either of the two alone. Hence, the subjectivity/objectivity relation could also be regarded as dependent, maybe of a more all-encompassing, as yet unknown third concept. Another option would be a dependency on the plurality relation with regard to the biomedical paradigm. However, the latter would rather be an effect of interpreting subjectivity and objectivity within a certain frame of understanding and hence represent a slippage towards plurality, not necessarily a dependence. Due to the possibility of standing alone without a necessary overlap with another concept relation (or concept for that matter), it is here deemed an independent relation between two different concepts of equal dignity. As these concepts are clearly connoted, the subjectivity/objectivity relation is subsumed under sub-section B.

Concerning future developments, plurality and quality can be linked to the so-called enhancement relation. When objectives and impacts are regarded crucial, enhancement effects may possibly be presented exclusively (Ryff, 2014). However, in the data, these presentations always appear to imply a focus on one of the two core concepts as more encompassing than the other (in Ryff, 2014, this is well-being). Enhancement thus emerges as future-oriented extension of either plurality or quality and hence a dependent relation (e.g., Jaberi et al., 2019; Kimiecik, 2011).

The plurality relation, moreover, constitutes the context in which the relation of well-being as positive health unfolds. As a positive side of health (i.e., well-being) presumes a negative side, which both then sum up to health in total, well-being can only represent a part of this complete health. Consequently, well-being as positive health is no self-sufficient relation but a dependent part of the plurality relation (e.g., Korp, 2016). It may also encompass notions of subjectivity and objectivity as connotations of (subjective positive) well-being and (objective) health (as in Burkert et al., 2015, for example) and may thus be understood as representing subjectivity/objectivity.

#### Slippage between congruence—complement—coincidence

The quality relation may contain certain leanings to merge with the congruence relation, due to the measurement instruments/questionnaires applied to determine the state of well-being. These may include a varying degree of “health” into the well-being umbrella, as Kahn and Juster (2002, p. 632) illustrate: “Virtually all studies that attempt to assess well-being include one or more questions about health”. The more health questions are used, the less well-being differs from health, but rather turns into it. Then, well-being and health become superimposable on each other, as is the characteristic in congruence.

Coming back to the subjectivity/objectivity relation: The presented states of subjective well-being and objective health are not as straightforward as is the case in plurality or quality (or enhancement). The relation between subjective well-being and objective health is rather characterized by a potential antagonism between those two sides, which may result in (more or less) conflict or confirmation concerning the level of subjective well-being in comparison to that of objective health. Conflict and confirmation may hence be called factual states of the relation (and effects concerning expectations of quality alignment in the subjectivity/objectivity relation). The two are highlighted in several textbooks (Brülde & Tengland, 2003; Hallberg, 2010; Hanson, 2004; Korp, 2016) but only a few articles (Bergland & Kirkevold, 2001; Brülde, 2000). As these effective states of the concept relation resemble those discussed in connection with the coincidence “relation”, a certain slippage between the subjectivity/objectivity relation and the coincidence relation appears to be implied and possible.

## Discussion

Since the days of Foucault, we know that knowledge is not neutral but constructs and is constructed in discourses (Foucault, 2002). As mentioned in the introduction, the health discourse is characterized by an abundance of normative expectations and possesses a powerful societal position while it simultaneously is marked by a great amount of insecurity and contradictions. The question is therefore what kind of normative power different understandings and choices of the concept relation possess under these discursive circumstances and what consequences that may imply.

### On the normativity of choosing sides, the “seemingly counter-intuitive” and the “regulars”

In this section, the normative potential and the resulting norm of the relation between health and well-being is discussed from a norm-critical stance (Björkman & Bromseth, 2019; Kumashiro, 2002; Pelters, 2018). That norm may reveal itself in the way a certain understanding of the concept relation is presented or is compared to the other understandings.

For a start, it should be emphasized that Korp (2016, referencing to Seedhouse 2004) is the only one presenting the notion that health and well-being could be completely unrelated concepts. As even the other comparative textbooks do not mention this relation, coincidence is considered possessing quite a minority status. Given the hegemonic notion of a conceptually important and intimate connection between health and wellbeing (e.g., Brülde, 2000; Kingma et al., 2011), assuming a non-relation may be considered a very basic non-normative, counter-intuitive version of the concept “relation”.

Departing from a more implicitly normative example in Brülde and Tengland (2003, p. 39), normative expectations regarding the relation of health and well-being begin to show. In the context of talking about “co-variations” of the two concepts, it says: “That every deterioration of a person’s well-being does not mean that her health deteriorates (and vice versa) does not exclude that many changes in the well-being dimension at the same time (by definition) constitute changes in the health dimension.” In this sentence structure, “not every” is contrasted to “many” and thereby describes the former as an exception of the latter, which represents the rule. That rule may include the exception, but it is still the rule and hence that which is judged as “common” and “expected” regarding the co-variation of health and well-being. In sum, the quote translates into an alignment between the quality of (objective) health and of (subjective) well-being, as “normal” co-variations. The exception of non-aligned qualities is accentuated and integrated into a hierarchy of expectations (see even Hanson, 2004), which may represent a way of turning the exception into a deviation (Björkman & Bromseth, 2019).

Another normative characteristic becomes obvious in the context of the subjectivity/objectivity relation, more precisely in how certain variations within the relation are presented. The “conceptual distinction between ‘experienced’ and ‘real’ health and ill-health” in Korp’s description of the health-cross (Korp, 2016, p. 61) presents one example. Here, the “experienced” subjective well-being is compared to an objective health that is labelled “real” and thus turns experience into something which is by contrast “not real” or at least not as real. Therefore, the hitherto presumed potential equality of subjectivity and objectivity (and health and well-being) may tip to one side. Drawing on the assumption of biomedicine as the hegemonic paradigm (Blaxter, 2010), that may render well-being inferior to health. It, moreover, points towards the hierarchy between scientific professionalism and mundane laity (Addelson, 2003) as cited and simultaneously reconstructed frame of reference. The subordination of well-being compared to health also becomes apparent in the quality and the plurality relation. In plurality, the greater significance or effectiveness of health as the focus concept is obvious. In studies focusing well-being/eudaimonia and the quality relation, an explanation of well-being and the recent move towards the use of the concept in health matters usually constitute some part of the introduction (see, e.g., Aldrich, 2011; Kimiecik, 2011; Martin & Woodgate, 2020). Such explanations or negotiations are, in contrast, regularly missing in health-related studies. Hence, the obvious need to explain and put well-being on the agenda confirms health as the established “norm”.

Moreover, health and well-being are not only expected to influence each other in an aligned fashion but to do so in a positive way, as the enhancement relation indicates. As a positive is the predominantly reported effect (with only one exception: Kahn & Juster, 2002) this might hint towards the necessity to deliver a positive message to argue for one’s point of view or, more profane, for publication of one’s study results. Although the interpretation of a mere positive impact cannot be discarded completely, the normative potential of the enhancement relation matches the notion of a constant optimizing in accordance with the narrative of people’s in principle unlimited health potential (Nettleton, 2013).

To sum up the picture portrayed in this section, the regular, normative understanding of the relation of health and wellbeing may be described as follows: Health and well-being are concepts that indeed have a relation. Their relation is characterized by a “quality alignment”, i.e., good health is combined to or includes a higher probability for good well-being and vice versa, so that the two concepts co-vary in a similar way. As the representative of “objectivity” and the more established concept in the health sector, health is regarded as the authority which takes the lead in determining that joint quality of health and well-being while well-being is positively connoted. Moreover, that aligned quality is assumed to be constantly increased as an expectation of an ever-developing health potential. The complementary irregular, counter-intuitive understanding consists then in health and well-being as not being related, with a quality that may not co-vary similarly but develop independently. Even if a relation were to be assumed, another counter-intuitive expectation would be that the quality of health and well-being may not develop towards betterment but stagnate or even decline.

### On the consequences of counter-intuitive relations for people’s understanding of health, disease, well-being and their connection with the health discourse

It has been shown that counter-intuition is linked to an unaligned, potentially contrary quality and quality development of health and well-being as well as to a “relation” called coincidence. In this section, I investigate how these conflicting and coincidental counter-intuitive, non-normative relations may impact on understandings of health, both in general and in particular (Franke, 2012; Blaxter, 2010; Brülde & Tengland, 2003; Herzlich, 1973, see theory section) and how this relates to the societal context described in the introduction. Generally, the counter-intuitive concept relation may present the threat of suspending all regularities concerning how health, disease and wellbeing may impact on each other and all boundaries between understandings of these concepts. To give an example, “health” may represent “disease” and what is supposed to be good for you (in terms of “healthy living”) may turn out to have bad consequences for one’s health, as is the case with orthorexia (Håman et al., 2016; Musolino et al., 2015).

Suspended regularities may also be stated regarding specific understandings of health. If *health is understood as the absence of disease*, it can be concluded that the clear dichotomy between the terms of health and disease may be questioned as different expressions of well-being need to be considered that may be able to bridge the gap between health and disease and question those boundaries. Hence, health may gain a “negative admixture” of disease (illness) and disease a “positive smack” of health (well-being) as indicated by the conflicts mentioned in descriptions of the health-cross (Brülde & Tengland, 2003; Hallberg, 2010; Hanson, 2004; Korp, 2016). Moreover, this implies that it is no longer clear which practices are labelled as matching and generating health or disease. This unmatching may limit people’s ability to predict and control the future and practice self-care (cf. Crawford, 2000) and increase health-related anxiety. The complication of one’s self-regulation and self-care, even self-fulfilment, may also arise in the *health as functionality* frame of understanding when a vagueness regarding the capabilities and competences required to this end becomes noticeable. This insecurity may impede people’s chances of becoming a functional private or public body, i.e., performing tasks to fulfil one’s (societal) role, reaching goals, and being a “normal” successful bio-citizen in healthistic neoliberal Western societies (Crawford, 2006; Halse, 2009). When health is understood as *balance*, the coincidence relation may complicate the evaluation and achievement of that balance. The very point of determining balance may be impeded as the points of reference for evaluating if a balance is obtained may or may not be correct and actions designed to establish balance may or may not be as beneficial or disadvantageous as expected. Achieving balance in this changing evaluative environment requires hence constant vigilance and preparedness to take compensatory actions with regard to the momentarily un-/balancing effects of a specific influence on “the whole picture”. While balancing may be a task even without coincidence, the novelty of the situation consists in the loss of reliable landmarks for the evaluation and achievement of balance. Therefore, the very idea of a possible harmony may become compromised, as may be the one of functionality in terms of capability and of disease as completely absent.

These potential conceptual challenges exist against the background of a western health discourse, which in itself is characterized by high complexity. Feelings of confusion, anxiety and uncontrollability are rising concerning the management and evaluation of health information beyond of what has been long discussed as an issue of lacking “health literacy” (e.g., Kristensen et al., 2016; Rangel et al., 2012). This effect is enhanced by the sheer amount of medially (e.g., online) consumable and personally (e.g., through wearables) obtainable health information (Alper, 2015) and even aggravated by the vast existence of medical dis- and misinformation (Ioannidis et al., 2017). Even social circumstances, expectations, priorities and feelings are perceived and evaluated in contradictory ways (e.g., Cameron et al., 2006; Pateman et al., 2016; Smith & Anderson, 2018), contributing to discursive complexity as well as personal (maybe even professional) perplexity on what is “right” or if there is a right at all. The health discourse and people’s decision-making, practice and understanding within these discursive boundaries are even more complicated by the ambivalent notion of health promotion as a practice implicitly demanding control and relieve simultaneously (Crawford, 2000). The simultaneousness in connection with the complexity of the health discourse renders it unclear if relieving or controlling activities actually are relieving or controlling anything and hence contribute to the promotion of health or not. This may contribute to coincidental, conflicting and contradictory discursive tendencies and add confusion. However, as the contemporary health discourse may be deemed as leading to conceptual confusion, conflict or coincidence as it is, the seemingly counter-intuitive understandings of the health-wellbeing relation and their consequences may rather be considered apparent indications of the complexity of the health discourse and its development instead of independent phenomena. In an even wider frame, these phenomena not least appear to represent the constant uncertainty about one’s state and status which is typical for dealing with knowledge in the second modernity (Giddens, 1991; Rose, 1996).

In consequence, there appears to be a good chance that the coincidental relation between health and well-being may against this discursive background very well be perceived as a rule rather than an exception. Moreover, when the option of a potentially conflicting or coincidental relation between health and wellbeing exists, the development of one’s health and well-being may be rendered quite unpredictable as the quality of health and well-being may or may not develop in an intended, aligned way, whatever the performed activity is (and whatever the amount of responsibility a person takes for it). All the proverbial 100-year-old smokers and drinkers and the people who come down with cancer at an early age despite living healthy may serve as examples (which I have encountered in interviews with people at risk for hereditary cancer during an earlier study, *reference will be included after acceptation*). In this fuzzy, ambiguous situation, health and wellbeing may well be framed as a new conceptual binary in addition to that of health and disease, as a way of raising awareness for the potentially ambivalent way of understanding and practicing well-being. That option appears to be more viable, compared to embedding an ambivalent well-being into the binary of health and disease, as wellbeing otherwise would need to be included into both health and disease.

### Implications of the spectrum of concept relations

As the highly important relation of health and well-being (Brülde, 2000; Kingma et al., 2011) permeates the health discourse in Western health societies (Kickbusch, 2007) in multiple ways, the implications of how the relation of health and well-being is understood are presumably many and exceedingly varied. Hence, focus is needed and will be mostly put on practical, ethical and normative implications, in general and connected to health education, with a “detour” past concept development.

In order to cope with the complexity of the concept relation, the first practical issue in need of tackling is to decide on whether to aim at developing the concepts (and its relation) or people’s ways of dealing with them. As much as conceptual development is desirable in order to create unambiguousness, that strategy may rather be expected to work in the longer run. The concept relation is far from unambiguously understood in medical philosophy (e.g., Brülde, 2000; Nordenfelt, 1987; Tengland, 2007), the health sector (e.g., www.who.int; Dooris et al., 2018) or society (e.g., Axelsson, 2015; Nettleton, 2013). That may impede scientific consensus on the one hand and the recapture of different existing understandings of relating health and well-being in the information society with its large capacity to store, yet significantly lower tendency to delete information (Webster, 2014), on the other hand. Both practical problems could exacerbate chances to narrow down intelligible options for understanding the concept relation. Moreover, a theoretical problem might need to be addressed. As mentioned, well-being may be regarded both belonging to the realm of health and that of disease in the health/disease binary. As this situation is challenging, two potential solutions are conceivable: one, to theoretically narrow down options for what is rightfully called well-being and two, to move from developing universally (or at least in the West) valid concepts to situated and context-sensitive concepts. As the former may turn descriptions into moral tools (something which Korp, 2016 problematizes in terms of “real” versus ‘fake well-being), the latter appears more attractive in societies that value equality and from the point of view of a medical ethics that emphasizes autonomy and justice (Beauchamp & Childress, 2019). That would, in effect, result in theorizing ambiguity and adjusting conceptual development to a diverse reality. In both cases, i.e., the development of concepts and of people, ambiguity and the uncertainty that comes with it needs to be faced in a way that not necessarily makes them disappear but manageable and appears as inevitably linked to an ethical standpoint.

The claim of a manageable uncertainty and ambiguity resonates with Giddens (1991) thoughts on personal requirements to strive towards the realization of one’s potential in an agentic, informed, reflective fashion despite one’s uncertainty about the future and the ambiguity of situations and knowledge (as exemplified by the various health-wellbeing-relations) in second modernity. Being able to manage and cope with ambiguity should hence be a key skill not least in questions of health and well-being, a skill whose acquisition should be a priority when the development of people is focused in health education and health promotion. Tolerance of ambiguity or uncertainty appears hence to be one of the most needed qualities for health decision makers, both professional and lay (i.e., all of us). That tolerance has been described as “the way an individual (or group) perceives and processes information about ambiguous situations or stimuli when confronted by an array of unfamiliar, complex or incongruent clues” (Furnham & Ribchester, 1995). Interpreting ambiguous situations as a source of threat or discomfort is regarded representing intolerance, while coping well in similar situations is deemed a sign of high tolerance towards ambiguity and uncertainty. This indicates an ability to perceive ambiguous situations as desirable and to take beneficial personal decisions despite an awareness of ambiguity (Budner, 1962). That ability appears to be a recommendable key skill when different conceptual understandings exist at the same time, rendering health and well-being into two potentially conflicting and/or coincidentally related concepts and impeding the task of “cultivating well-being and maintaining health” (Grundmann, 2014, p. 552) by the fuzziness of competing information, authorities and demands in the present-day health discourse (cf. Kristensen et al., 2016).

Tolerance of ambiguity requires, however, a conscious understanding of the very ambiguity, which is to be tolerated, and the topic it concerns. Coming back to Paulo Freire (1973), raising this critical awareness (“conscientisation”) may be initiated by discussing and problematizing in order to achieve change. In this context, it is suggested that the overview provided in this paper (which may hopefully find its way into educational course literature) may prompt such dialogic processes as a reflective tool, not as a given morsel of knowledge (as that would merely represent the banking model, which educates by filling people’s supposedly empty heads with pre-designed facts, Freire, 1973). In doing so, it is assumed that not only the pedagogic goal of picking up on and proceeding from wherever the student stands will be met. Even normative expectations, implicit assumptions and experiential challenges concerning the relation of health and well-being may emerge if the responsibility for change is disseminated to a wide variety of involved agents (as mentioned in the introductory section) and the productive power of concept relations become apparent. That would be the switch from critical to norm-critical education (Björkman & Bromseth, 2019; Pelters, 2018) that exceeds bio-pedagogics as a means of securing biomedical hegemony and biopolitics (Harwood, 2009) and aims for broadening the normative spectrum and create conditions for tolerating ambiguity. Here, the political and at the same time ethical potential of a (norm-)critical health education becomes apparent as it traditionally aims for the promotion of societal participation instead of conformity (Freire, 1973). Moreover, tolerance of ambiguity has proven to diminish tendencies to resort to stereotyping and categorizing as a means to reduce uncertainty (Valutis, 2015), is associated with a relativist approach to ethical principles in which ethical judgements tend to be situated and contextualized instead of principle-driven (Yurtsever, 2000) and is based on an ethics of ambiguity characterized by the virtue of respect (Domen, 2016). Educating health professionals to-be in a way that promotes ambiguity tolerance is therefore assumed to be beneficial for students’ future working life (ibid.) and also reflect Beauchamp and Childress (2019) fundamental ethical principles: autonomy, non-maleficence, beneficence and justice concerning the promotion of diversity and respect.

When turning to consequences of specific versions of the congruent, complementary and coincident health-wellbeing-relations, different aspects of normative expectations are used as vanishing points for a discussion of their implications. More precisely, the consequences of a) a quality-aligned health-wellbeing-relation as a promise of controllability and feasibility, b) the performance character of an ever-enhancing health and well-being as a justice-related challenge and c) the positive connotation of well-being as an enhancer of confusion will be focused.

Concerning a: The different versions of the health-wellbeing-relation have to be viewed within a spectrum moving from homogeneity (congruence) to heterogeneity (coincidence), which implies a move from clarity to fuzziness and security to insecurity but also predetermination to freedom. Within this spectrum, a quality-aligned health-wellbeing-relation (as existing in congruence and complement) may be regarded as a promise of individual controllability and feasibility. As such, the promise matches hegemonic traits of the health discourse (Ayo, 2012; Crawford, 2006; Metzl, 2010) and provides a good example of discursive (re-)construction. More importantly, assuming that health and well-being can be produced simultaneously or in a mutually enhancing way may motivate what is understood as “healthy living” as a way to achieve both health and well-being. This motivation may be based on assuming self-efficacy, as described, e.g., in the health belief model (Rosenstock et al., 1988). But what happens if these expectations are not met, e.g., by being diagnosed with a disease despite healthy living or by not experiencing well-being in combination with health or health-related activities (cf. a pod about “gym-haters”, https://sverigesradio.se/avsnitt/1026371)? How much discrepancy may be accepted until frustration, doubts and/or feelings of a loss of control replace self-referred trust and hopes for experiencing health and well-being (cf. Pelters & Wijma, 2016)? It is the assumption of a regularity (with the label of objectivity adding dignity to it), which makes differing developments appear as exceptional or even deviating, rendering diminished or declining well-being and health (mostly) unacceptable instead of (sometimes) unavoidable. Here, the coincidence relation may actually own a potential for reassessing the insecurity and uncontrollability often associated with a lack of consistency as is undoubtedly significant in just coincidence. That “gap” may instead be understood as a higher degree of freedom and as such enable a more pragmatic approach to generating both health and well-being that benefits from being de-coupled. One example is provided in the mentioned pod, which presents one “gym-hater” as a person who has given up on experiencing well-being in connection to exercise and instead regards training a duty like brushing teeth (https://sverigesradio.se/avsnitt/1026371). Another possible example would be to indulge in pleasures labelled as “hedonistic well-being” without experiencing a bad conscience, i.e., without judging that sort of well-being as (morally) wrong. While this is the upside, the downside may include a potential for a diminished physical population health and the loss of stigmatization and shame as motivational approaches in health promotion (albeit the effectiveness of the approach is somewhat inconclusive, see, e.g., Amonini et al., 2015; Kim et al., 2018).

Concerning b: The enhancement relation suggests that health and well-being may be ever-enhancing, turning their improvement into something that appears to be depending on and achievable by performance. That enhancement-oriented achievement may be attributed the character of a moral duty for healthy bio-citizens (Halse, 2009). However, regarding health a purchasable commodity in the market place in the face of the unequal distribution of health due to resource allocations and the dissemination of different forms of capital (Nettleton, 2013; Pateman et al., 2016; Scrambler, 2012) indicate the impact of societal positions on people’s seemingly equal options to improve health and well-being in a mutual process. The norm of an expected constant upgrading indicates hence issues of status and privileges connected to the health-wellbeing relation, with regard to people’s chances to succeed in its realization. This justice-related challenge, moreover, may be aggravated as non-achievement may result into stereotyping and in the longer run stigma, which then again may enhance health inequalities (Hatzenbuehler et al., 2013). Again, the coincidence relation may stand for decreased expectations and increased options to act, think and feel in accordance with the resources, which are available for a specific person in a certain milieu. That may provide a way past resistance (Broom, 2008) and misunderstandings (Cameron et al., 2006) in health education interventions. The included dilemma, however, resembles the above mentioned motivational problematic, not only with regard to striving for personal development but also concerning the question of responsibility for people’s health, i.e., personal responsibility versus the responsibility of the welfare state (see, e.g., Vithus et al., 2018) as the price of freedom.

Last but not least, the positive connotation of well-being may function as a confusion enhancer as well-being owns a positive connotation, despite the co-existence of hedonic and eudaimonic understandings as different ways to interpret well-being (Ryan & Deci, 2001; Tončić & Anić, 2015). Understanding well-being eudaimonicly may support a normative quality alignment between health and well-being and support congruent and complementary concept relations. The hedonistic version, in contrast, rather leads to a “quality decoupling” in which well-being and health may develop in contradictory directions (or not), which might resonate more with a coincidental concept relation. As both are, however, usually indiscriminately addressed as “well-being”, the positive connotation of well-being may support the perceptions of confusion and uncertainty and/or choosing an understanding moulded to one’s liking.

## Method discussion and suggestions for further research

Due to the small scale of the study and its qualitative design, it is possible that a version of the relation between and well-being has been missed. Especially, the approach of choosing course literature as primary data sources and scientific texts from databases as well as online publications of the WHO and other, non-scientific online texts only as secondary data sources may be regarded as presenting a very special and potentially limiting perspective on the question of relating health and well-being. Combining more and different search terms, continuing the search in a number of other databases or discussing more and different definitions of health and well-being in detail may extend the range of covered versions and add complexity and depth to their description. To include more empirical data in which health and well-being are investigated from different stakeholders’ standpoints may also refine the descriptions of the mentioned relations.

However, the presented combination of analytic methods, the range of different data sources and the two collection rounds are considered appropriate to allow for the study to cover at least most versions of the health-wellbeing relation as socially accepted practices. Hence, the study is assumed to provide the comprehensive, structurally generalized overview of the different concept relations that it aimed for. Applying a notion of structural generalization (Oevermann, 2002), it is deemed appropriate to focus attention on the different versions of the relation between health and well-being, while concurrently diminishing the need to collect and review a big body of data material as similarities can be neglected, in favour of differences. That is considered providing a justification for the small scale of the review study and for its potential to map the relation between health and well-being.

As this study aims for identifying the status quo of interpreting the relation of health and well-being and its consequences, a completely different take on researching this relation would be working for a definition of the relation. This is suggested as another question for further (philosophical) research and would contribute to more clarity concerning how the health-wellbeing relation should be conceptualized. Even if the different versions of the relation mentioned in this paper would still be “out there” in society, a conceptual clarification may diminish the spectrum of accepted interpretations in the longer run.

## Conclusion

A congruence, a complement and a coincidence relation between health and well-being with different independent and dependent versions could be described, whereas congruently or complementarily related health and well-being mostly appear to confirm existing understandings of health and well-being and show a quality alignment, their quality is potentially decoupled and health understandings questioned if they share a coincidental or conflicting relation. Despite the match between quality-aligned versions of the concept relations and hegemonic characteristics of the present-day health discourse in western societies, the coincident relation may be easily perceived as a rule rather than an exception due to discursive complexity.

Health and well-being stand thus “right by each other’s side”, yet in a messy, complex fashion, not least because their relation tends to ambiguous coincidence in second modernity. A desire of control may arise in the face of ambiguity and developing a tolerance towards that ambiguity is deemed advisable as is a clarification of the applied health-wellbeing relation.

## Supplementary Material

Supplemental Material
