# Supplementary material for: Right by your side? – the relational scope of health and wellbeing as congruence, complement and coincidence
Source: Int J Qual Stud Health Well-being. 2021 Jun 7;16(1):1927482. doi: 10.1080/17482631.2021.1927482 (PMC8204984; doi:10.1080/17482631.2021.1927482)
Supplement: Supplemental Material [file ZQHW_A_1927482_SM2095.zip › Supplementary files/Supplement data_collection_selection.docx]

Collection and selection of primary and secondary data

Primary data:

*Course literature* includes books and articles which are used as introductory, first-term course literature in two Swedish bachelor programs in health education, a term during which basic concepts are in focus. Publications included in the search represented a) comprehensive introductory texts to b) theories of health, public health, health promotion, health identities and health education which c) introduced the concepts of health and well-being. Exclusion criteria were literature on a) specific topics (for example empowerment or obesity), b) epidemiology (when not explicitly dealing with issues of health and well-being) or c) methods and methodology.

In collection round no. 1, four of the 18 publications on the two lists were selected after checking for matching the eligibility criteria by title, table of contents and subject index and appearance of the term well-being/wellbeing/well being (please note that all data searches were conducted with all dictions, even if not mentioned explicitly). One more book – labelled as “to be included in next year’s course” by the responsible lecturer – was added by recommendation. A total of five textbooks (all Swedish) presented the final sample for understanding which relations were marked important, sound and established enough to be presented to first-term students. The sample contains one conceptual investigation on health and disease, one introduction to basic public health, one introduction to theoretical and practical health promotion and public health, one introduction to health promotion in working life and one book on health and lifestyle. The described concept relations were named and outlined.

In round no. 2, four of the 29 publications on the four lists were selected, two textbooks and two articles (one in English, three in Swedish) represented the final sample, which included conceptual investigations and introductions regarding health, health promotion and health identities. All established relations were confirmed and could be illustrated further. Moreover, one new relation was reconstructed.

Secondary data

Secondary data has been chosen as a consolidation of the findings from the first stage, but also for adding potentially missed cases. It was inspired by a scoping review strategy as “a process of mapping the existing literature or evidence base” (Armstrong et al. 2011: 147) for available key concepts and research findings in a rapid process. Compared to a systematic review, the scoping approach uses broader research questions, a more qualitative synthesis and is not limited to high quality research outcomes. Those attributes have been realized by a) not defining the central concepts health and well-being, b) using post-hoc definitions of exclusion and inclusion criteria for data collection, c) scoping three different (additional) data sources which are not limited to research literature and d) combing the scoping approach with a hermeneutical analysis. In doing so, the data collection focused on breadth (Arksey and O’Malley 2005) even if it was limited to a small scale for reasons of time. The secondary data search was performed at scientific databases as well as the WHO (as an indirect stakeholder consultation, cf. ibid.) and Google (as indirect “common people” stakeholder consultation on the World Wide Web, cf. ibid). The screening process was adjusted to the specific search purpose and target data source of the collection step in question. Exemplary evidence from scientific literature has been added while writing to illustrate and confirm the spectrum in this article.

*Databases: scientific literature*

During the two data collections, three databases each were searched to understand the state of research and scientific discussion regarding the relation between health and well-being. During the first collection, Pubmed, Web of Science and OneSearch were searched, whereas in collection round no. 2, Academic Search Premier replaced OneSearch, due to accessibility issues. During both data collections, previously established relations were confirmed and illustrated, although no new relations were reconstructed.

In data collection round no. 1, databases have been searched using the following search terms: “relation between health and well-being”; “relation between well-being and health”; “connection between health and well-being”; “connection between well-being and health” as well as “health AND well-being AND ‘concept relation’”. Included were publications discussing the concepts health and well-being theoretically, excluded were mere empirical studies. In a first round all records (n = 793) were screened by title for meeting the study scope. 213 duplicates were excluded. In a second stage, abstracts of the remaining 43 records were read to determine if the record was in scope with the study. On this stage, empirical studies and those whose theoretical discussion was deemed insufficient were excluded. On the last stage, the remaining papers were assessed/included based on a full-text reading and the one remaining book’s text was read to determine relevant chapters. This final sample (n = 5) contained one practical viewpoint discussing the health concept, one review of the concept of well-being discussing its link to health and different areas of life, a commentary on the challenges to the spirit of WHO’s Ottawa Charter discussing disabled persons rights, one conference report presenting a workshop on concepts of health and disease and one doctoral thesis discussing health as a human right.

In data collection round no. 2, the following keywords were used (summarized here as a Boolean search mode): i) Health AND ii) “well-being” Or “well being” OR “wellbeing” AND iii) “concept analysis” OR “concept relation” OR “concept definition” OR “theoretical concept”. Inclusion and exclusion criteria were kept, while only peer reviewed scientific articles were included to boost the number of scientific contributions in the study, which have been somewhat disregarded during the first data collection. In a first round all records (n = 625) were screened by title for meeting the study scope. 161 duplicates were excluded. In a second stage, abstracts of the remaining 51 records were read to determine their scope equivalence. On the last stage, the remaining papers were assessed/included based on a full-text reading. This final sample (n = 17) contained four studies using empirical input for a theoretical aim, three critical discussions of practices and ten concept analysis and discussions.

*WHO: indirect stakeholder consultation*

The internet presentation of the WHO was searched for texts linking health and well-being in order to understand if the most important stakeholder on the health market and provider of one of the most cited health definitions conceptualizes the health-well-being relation in an additional way. The site www.who.int has been searched using the search terms “‘health’ AND ‘well-being’". Included were all content and all formats on the global sites on www.who.int, excluded were all regional sites as the relation of health and well-being is regarded being of overarching interest. Of the 1730+1100+1100 initially found hits in connection to the different spelling of well-being, 1100 were discarded due to duplication in a first screening step. In a second step, a scope of the first 100 of the 1730 respectively 1100 hits were reviewed and assessed for new ways of interlinking health and well-being by screening the title and text excerpts listed as search results. If the information proved insufficient to do so, the whole website was called. At this stage, ten of the 200 hits were selected as probably describing a new relation. Among those sites were publications, events and fact-sheets. However, no new relations were found after reviewing the hits as a whole.

*Google search engine: indirect online “common people” stakeholder consultation*

A Google-search for the relation between well-being and two major health promoting practices, i.e. determinants of health (food and exercise) has been performed. The search was performed in Swedish using the search terms “well-being + food” (välbefinnande + mat) and “well-being + exercise” (välbefinnande + träning). Targeting food and exercise is conditioned by their high status as *the* two most relevant health-promoting practices emerging from the so-called obesity crises (Quennerstedt et al., 2010) and as such appear as valid representatives of health. This notion is confirmed by studies on lay persons’ understandings of health (Blaxter 2010; Flick 2000) in which health has been described as a lifestyle and the practice of living healthfully. 454 000 hits were found for the combination of well-being and food, 239 000 hits for the exercise combination. All types of web resources have been included and a scope of the first 100 of each set were reviewed and assessed for new cases of health-well-being relations. The list of relations derived from text books or data base search were used as exclusion criteria. If the information provided by Google proved insufficient to do so, the whole website was called. Eight hits could be identified as possibly containing new forms of interlinkage and these websites were reviewed as a whole. Among these were website of county councils, health bloggers, companies offering health promotive activities and health magazines. One new case of relation could finally be identified on a website that belonged to a health magazine.
