# Supplementary material for: Right by your side? – the relational scope of health and wellbeing as congruence, complement and coincidence
Source: Int J Qual Stud Health Well-being. 2021 Jun 7;16(1):1927482. doi: 10.1080/17482631.2021.1927482 (PMC8204984; doi:10.1080/17482631.2021.1927482)
Supplement: Supplemental Material [file ZQHW_A_1927482_SM2095.zip › Supplementary files/Table_supplement_data overview data_and_relations.docx]

**Table 3:** **Overview of data and depicted relations (in italics: results of data collection no. 2)**

| Publication: author & title | Congruence | Complement | Coincidence |
| --- | --- | --- | --- |
| **Teaching materials (Textbooks & articles)** |  |  |  |
| Brülde B. & Tengland P.A. (2003): Hälsa och sjukdom – en begreppslig utredning. [Health and disease – a conceptual investigation] | X | Plurality  Quality  Subjectivity/objectivity: confirming/conflicting--- Enhancement: bidirectional | --- |
| Hallberg L. (2010): Hälsa och livsstil. Forskning och praktiska tillämpningar. [Health and Lifestyle. Research and implementations] | X | Plurality  Quality  Subjectivity/objectivity: confirming/conflicting--- | --- |
| Hanson A. (2004): Hälsopromotion i arbetslivet. [Health promotion in the working life] | X | Plurality  Enhancement: bidirectional  Subjectivity/objectivity: confirming/conflicting--- | --- |
| Pellmer K., Wramner B. & Wramner H. (2017): Grundläggande folkhälsovetenskap.[Basic public health] | X | Plurality |  |
| Scriven A. (2013): Ewles & Simnett Hälsoarbete. [Ewles & Simnett promoting health] | X | Plurality |  |
| *James, A. & Hockey, J. (2007): Embodying health identities.* |  | *Subjectivity/objectivity* |  |
| *Korp, P. (2016): Vad är hälsopromotion? [What is health promotion?]* | *X* | *Plurality*  *Well-being = positive health*  *Quality*  *Subjectivity/objectivity: confirming/conflicting---* | *X: opening for conflict + compatibility but*  *inferior concern*  *---* |
| *Quennerstedt, M. (2007): Hälsa eller inte hälsa – är det frågan? [Health or no health – is that the question?]* |  | *Subjectivity/objectivity*  *Enhancement: unidirectional (wb=>h)* |  |
| *Vilhelmsson, A. (2014): Psykisk ohälsa, folkhälsa och medikalisering: har det egentligen någon betydelse vilket hälsobegrepp som används? [Mental ill health, public health and medicalization: does it really matter what concept of health is used?]* | *X* | *Subjectivity/objectivity*  *Plurality* |  |
| **Data base search** | | | |
| Kingma E., Chisnall B. & McCabe M. (2011): Interdisciplinary workshop on concepts of health and disease: report. |  | Subjectivity/objectivity  Quality |  |
| Erk Ch. (2011): Health, Rights and Dignity: Philosophical Reflections on an Alleged Human Right. (book) | X | Quality  Plurality |  |
| Wolbring G. (2006): Three Challenges to the Ottawa Spirit of Health Promotion, Trends in Global Health, and Disabled People |  | Quality  Plurality |  |
| Brülde B. (2000): On how to define the concept of health: A loose comparative approach. |  | Plurality  Subjectivity/objectivity: confirming/conflicting--- | --- |
| Ryff C.D. (2014): Psychological Well-Being Revisited: Advances in the Science and Practice of Eudaimonia. |  | Enhancement: bidirectional |  |
| *Boers, M. & Cruz Jentoft, A.J. (2015): A new concept of health can improve the definition of frailty.* | *X* | *Plurality* |  |
| *Pons-Vigués M., Berenguera A., Coma-Auli N., Pombo-Ramos H., March S., Asensio-Martínez A., Moreno-Peral P., Mora-Simón S., Martínez-Andrés M., Pujol-Ribera, E. (2017): Health-care users, key community informants and primary health care workers' views on health, health promotion, health assets and deficits: qualitative study in seven Spanish regions.* | *X* | *Plurality* |  |
| *Sointu E. (2006): The search for wellbeing in alternative and complementary health practices.* | *X* | *Quality*  *Subjectivity/objectivity (traces)* |  |
| *Dooris M., Farrier A., Froggett L. (2017): Wellbeing: the challenge of 'operationalising' a holistic concept within a reductionist public health programme.* | *X* | *Quality* |  |
| *Svalastog, A.L., Donev, D., Kristoffersen, N.J., Gajović, S. (2017): Concepts and definitions of health and health-related values in the knowledge landscapes of the digital society.* | *X* | *Plurality* |  |
| *Burkert, N. T.; Rami, R.; Beier, N.; Freidl, W. (2015): Differentiating health statuses using positive health indicators in an occupational context.* |  | *Plurality*  *Subjectivity/objectivity*  *Well-being = positive health (traces)* |  |
| *Aldrich, R.M. (2011): A review and critique of well-being in occupational therapy and occupational science* | *X* | *Quality*  *Well-being = positive health (traces)* |  |
| *Konu, A., Rimpelä, M. (2002): Well-being in schools: a conceptual model.* |  | *Quality* |  |
| *Martin, K.M., Woodgate, R.L. (2020): Concept analysis: the holistic nature of sexual well-being.* | *X* | *Quality* |  |
| *Modise, L., Johannes, M.: Well-Being and Wellness in the Twenty-First Century: A Theanthropocosmic Approach.* | *X* |  |  |
| *Agenor, C., Conner, N., & Aroian, K. (2017): Flourishing: An Evolutionary Concept Analysis* | *X* | *Subjectivity/objectivity*  *Enhancement: unidirectional (wb=>h)*  *Flourishing (well-being) as positive health* |  |
| *Bergland, Å. & Kirkevold, M. (2001): Thriving – a useful theoretical perspective to capture the experience of well-being among frail elderly in nursing homes?* |  | *Subjectivity/objectivity: confirming/conflicting---*  *Thriving (well-being) as positive health* | *---* |
| *Jaberi, A., Momennasab, M., Yektatalab, S., Ebadi, A., & Cheraghi, M. A. (2019).: Spiritual Health: A Concept Analysis.* | *X* | *Enhancement: bidirectional*  *Subjectivity/objectivity*  *Plurality* |  |
| *Kimiecik, J. (2011): Exploring the Promise of Eudaimonic Well-Being Within the Practice of Health Promotion: The “How” is as Important as the “What.”* |  | *Quality*  *Enhancement: bidirectional*  *Subjectivity/objectivity* |  |
| *Kahn, R. L., & Juster, F. T. (2002): Well–Being: Concepts and Measures.* |  | *Quality*  *Subjectivity/objectivity (traces)*  *Well-being as positive health (traces)* |  |
| *McLeod, J., & Wright, K. (2016): What does wellbeing do? An approach to defamiliarize keywords in youth studies.* | *X* | *Well-being = positive health (traces)* |  |
| *Grundmann, C.H. (2014): To Have Life, and Have It Abundantly! Health and Well-Being in Biblical Perspective* | *X* | *Subjectivity/objectivity (traces)* |  |
| **WHO** | | | |
| www.who.int | X | Plurality  Quality  Enhancement: bidirectional |  |
| **Different stakeholders’ web presence** | | | |
| Google-search |  | Quality  Plurality  Enhancement: bidirectional  Subjectivity/objectivity | Compatibility  Conflict |
